# Winter Cover Cropping in Sustainable Production Systems: Effects on Soybean and Synergistic Implications for Rhizosphere Microorganisms

**DOI:** 10.3390/plants13213091

**Published:** 2024-11-02

**Authors:** Marjana Vasiljević, Srđan Šeremešić, Dragana Miljaković, Vuk Đorđević, Jelena Marinković, Bojan Vojnov, Vladimir Aćin

**Affiliations:** 1 Legumes Department, Institute of Field and Vegetable Crops, 21000 Novi Sad, Serbia; dragana.bjelic@ifvcns.ns.ac.rs (D.M.); vuk.djordjevic@ifvcns.ns.ac.rs (V.Đ.); jelena.marinkovic@ifvcns.ns.ac.rs (J.M.); 2 Department of Field and Vegetable Crops, Faculty of Agriculture, University of Novi Sad, 21000 Novi Sad, Serbia; srdjan.seremesic@polj.uns.ac.rs (S.Š.); bojan.vojnov@polj.uns.ac.rs (B.V.); 3 Small Grain Department, Institute of Field and Vegetable Crops, 21000 Novi Sad, Serbia; vladimir.acin@ifvcns.ns.ac.rs

**Keywords:** sustainable cropping systems, cover crops, *Glycine max*, organic production, low-input production

## Abstract

The aim of this study was to evaluate the effects of winter cover crops (CCs) on soybean agronomic performance and their implications for different physiological groups of rhizosphere microorganisms in two sustainable production systems. The production techniques for rye, peas, and oats are well known, but their suitability as CCs for soybean (organic and low-input) production needs to be examined. After two years of trials, soybean yields among the two tested winter CCs (peas and oats (P + O) and rye (R)) were statistically significant only for P + O. The soybean yield in succession to P + O as winter CCs was 3.0 t ha^−1^, whereas in succession to R, it was 2.7 t ha^−1^, and in the control plot, it was 2.6 t ha^−1^. The average soybean grain protein content was in the range of 40 to 41% dry matter (DM), while the oil content ranged from 20 to 22% DM. Protein and oil content primarily depends on the selected soybean variety and it is confirmed through this study that, in the studied system, we can obtain adequate grain nutritional quality. The results indicate an increase in the abundance of total bacteria, ammonifiers, and free N_2_-fixing bacteria in the rhizosphere, depending on the selected CCs, and differences between the tested production systems. According to this study, winter cover crops (CCs), including peas and oats (P + O) and rye (R), can be included in crop rotation for soybean. CCs can be the answer to agro-biodiversity empowerment in less diverse soybean cropping systems, along with other benefits that CCs can provide at the level of crop rotation. In addition, in almost all aspects of the study, organic production was ahead of low input. Low input is an adequate production system if there are no opportunities for organic certification and for producers who are aware of the advantages of sustainable systems, and it can also represent a transitional path towards regenerative agriculture or organic production.

## 1. Introduction

Dilemmas related to food safety in the context of quality and quantity, environmental challenges, and lack of resources are changing the perspective on production systems [1]. The foundation of conventional agriculture comprises the overuse of inputs, disturbance of ecological balance, and deterioration of natural resources [2]. Significant improvements are needed in cultivation technology to make the shift from conventional cultivation systems to sustainable production systems [3]. Legumes, including soybeans, have been particularly important in crop rotation because of their role in soil conservation and provision of multiple agro-ecosystem services [4]. Soybean (*Glycine max* (L.) Merr.) has great economic and agronomic importance arising from its versatile usage [5,6]. Globally, soybean-growing areas (144 million ha) provide around 370 million tons of grain each year [7]. Due to its favorable grain chemical composition (about 40% protein and about 20% oil), soybean is used in feed and food, pharmaceuticals, and other industries [8,9,10]. According to [11], contemporary technologies are needed to increase the productivity of soybean without degradation of the environment since it is one of the most widely grown field crops in the world. The advantages of soybean production are mostly due to its nitrogen fixation ability, which helps to reduce the use of nitrogen fertilizers for its production and for the next crop [12]. This coincides with numerous studies on the role of soybeans and their interactions with practices of various alternative and sustainable cropping systems, e.g., organic or low-input production. Sustainable agriculture aims to balance the economic, environmental, and social aspects of production, creating the preconditions for systems that are resilient and stable in the long term [13]. Currently, organic production is considered a key link in a sustainable agricultural system, where the application of its methods leads to the conservation of the ecosystem and human health, as well as the preservation of autochthonous and wild varieties [14]. Under the umbrella of national and EU regulation, the basic characteristic and advantage of organic production is the preservation of biodiversity for the mutual benefit of both humans and nature, and recognition of it by end users [15]. Ref. [3] states that low-input technology requires the adoption of the most basic cultivation practices, including reduced cultivation, less mineral fertilizer use, and the application of preparations to increase soil microbiological activity. According to [16], the use of on-farm resources is the defining characteristic of low-input farming systems, sustainable agriculture principles, and soil conservation practices, and cover-cropping systems have been promoted as alternative practices to soybean monoculture [17]. Low-input production is not widespread in practice due to heterogeneity and diversification requests. The proposed practices for field crops include decreasing tillage/no tillage and using cover crops (CCs), increasing the frequency of perennial crops in crop rotations, agroforestry, converting marginal lands that are poorly suited for annual cropping to perennial, and the inclusion of buffer strips [18,19].

The introduction of CCs into crop rotation can amplify the beneficial effects related to natural resource preservation, primarily soil, while increasing profits and leading to many other benefits [20]. However, studies on cover crop adoption for soybean are scarce and rarely provide sufficient information on the options for potential CCs in the improvement of soybean cropping practices. Before choosing a winter CC for soybean, it is important to consider the cultivation system, location, soil type, input costs, and the timing of their establishment and termination [21]. In order to maximize the positive effects of CCs, it is important to consider proper mechanical measures for the termination of CCs [22] and to avoid the use of chemical products. Due to the complex impact and length of the growing season of CCs and meeting N demands, it is sometimes difficult to interpret the interaction between soybeans and CCs, so it is necessary to consider all benefits and factors leading to increased soybean yields [23]. However, the grain yield and protein content of soybeans can also depend on the genetic basis, agro-ecological conditions, type of production system, and applied cultivation practices. The soil quality and functioning of an agro-ecosystem highly depend on the soil microbial decomposition activity and the regulation of many biogeochemical processes [24]. According to Ref. [25], it is necessary to investigate the role of biodiversity in ecosystem function and its relationship with other ecosystem services on the level of crop rotation. Understanding the relationship between microbial diversity distribution and ecosystem functioning is crucial to comprehend how ecosystems react to environmental changes [26]. Therefore, soil microorganisms are an integral part of soybean observation in sustainable production systems. The objectives of this study were to investigate (a) soybean yield; (b) grain quality, in particular protein and oil contents; and (c) the response of rhizosphere microorganisms and their synergism to the introduction of a cover crop in soybean under two different production systems, low-input and organic.

## 2. Results

### 2.1. Soybean Yield

**Figure 1:**
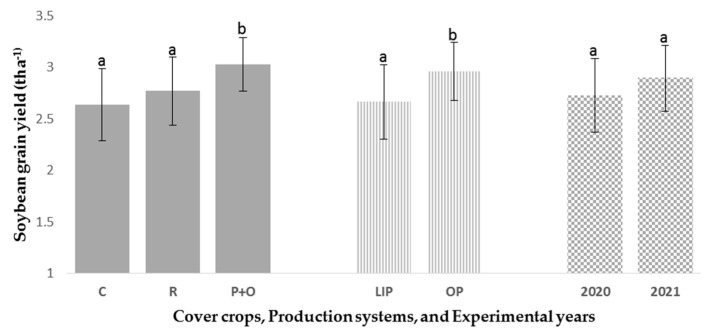
Average soybean grain yield (t ha^−1^) for two-year trials using different CCs and production systems. C—control; R—rye; P + O—peas and oats; LIP—low-input production; OP—organic production; 2020 and 2021—experimental years. Error bars show standard deviation, and letters on top of error bars indicate no significant difference at *p* < 0.05 using Tukey’s HSD test.

Across the years and production systems studied, the average soybean yield was 2.8 t ha^−1^, which is comparable to the average soybean yield in conventional production in Serbia (2.9 t ha^−1^) from 2019 to 2021. The soybean yield in succession to P + O as a winter CC was 3.0 t ha^−1^, whereas in succession to R as a CC, the yield was 2.7 t ha^−1^, and in the control, the soybean yield was 2.6 t ha^−1^. There were significantly higher soybean yields in OP systems (Figure 1). In addition, the year made a significant difference, as did the interaction between the production system and year (Table 1). Although two soybean varieties were tested in this experiment, only average values were presented because there was no difference between them and this provided a clearer view of the results obtained.

### 2.2. Soybean Height and Biomass

**Figure 2:**
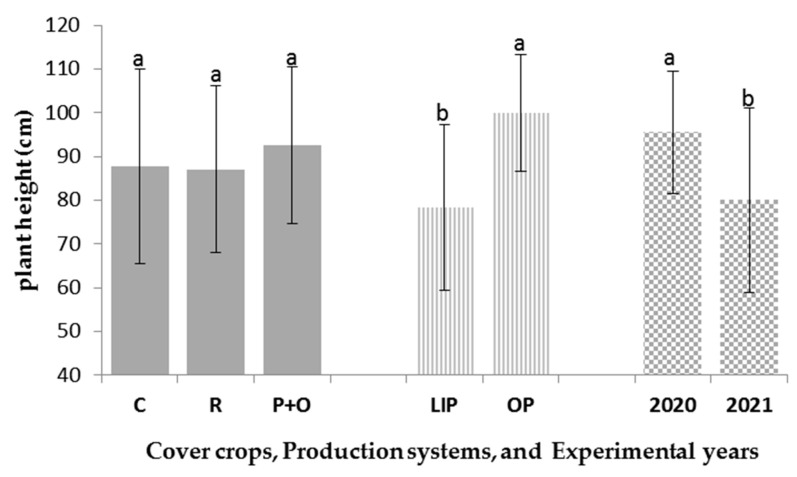
Average soybean plant height (cm) for two-year trials using different CCs and production systems. C—control; R—rye; P + O—peas and oats; LIP—low-input production; OP—organic production; 2020 and 2021—experimental years. Error bars show standard deviation, and letters on top of error bars indicate no significant difference at *p* < 0.05 using Tukey’s HSD test.

**Figure 3:**
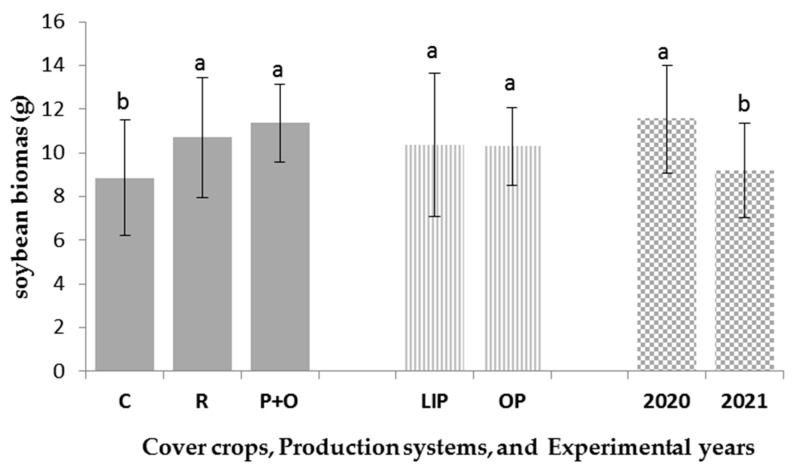
Average soybean plant biomass (g) for two-year trials using different CCs and production systems. C—control; R—rye; P + O—peas and oats; LIP—low-input production; OP—organic production; 2020 and 2021—experimental years. Error bars show standard deviation, and letters on top of error bars indicate no significant difference at *p* < 0.05 using Tukey’s HSD test.

It was observed that the average soybean plant height (cm) was in the range of 53.8 to 108.6 cm, and the highest value of soybean plant height was for P + O at 92.6 cm (Figure 2). Soybean biomass average value was in the range from 8.9 to 11.4 g, and the highest values were noted for P + O at 11.4 g per plant (Figure 3).

### 2.3. Soybean Grain Protein and Oil

**Figure 4:**
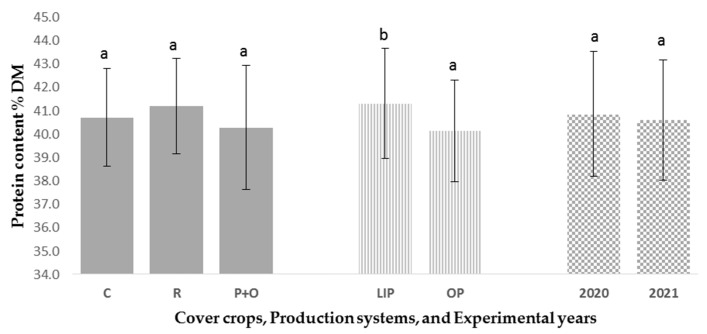
The average protein content of soybean in two-year trials using different CCs and production systems. C—control; R—rye; P + O—peas and oats; LIP—low-input production; OP—organic production; 2020 and 2021—experimental years. Error bars show standard deviation, and letters on top of error bars indicate no significant difference at *p* < 0.05 using Tukey’s HSD test.

**Figure 5:**
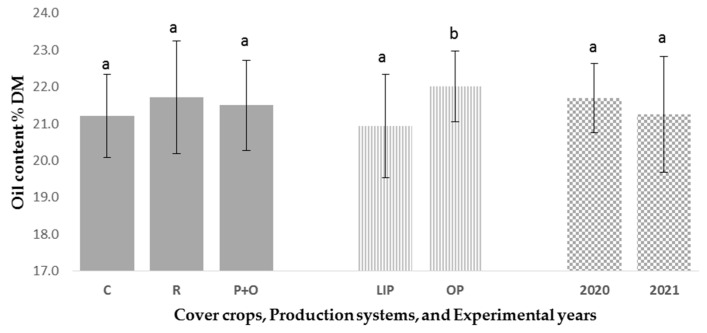
Average oil content of soybean in two-year trials using different CCs and production systems. C—control; R—rye; P + O—peas and oats; LIP—low-input production; OP—organic production; 2020 and 2021—experimental years. Error bars show standard deviation, and letters on top of error bars indicate no significant difference at *p* < 0.05 of Tukey’s HSD test.

In this study, the average soybean grain protein content was in the range of 40.2 to 41.1% of dry matter (DM), while the oil content ranged from 20 to 22% DM. Our analysis revealed a higher protein content in the LIP and a higher oil content in the OP (Figure 4 and Figure 5). No significant differences were observed in the protein or oil contents between the CC treatments (Figure 4 and Figure 5), but there were differences between the production systems and between the interactions between the production systems and years (Table 2).

### 2.4. Microorganisms in Soybean Rhizosphere

**Figure 6:**
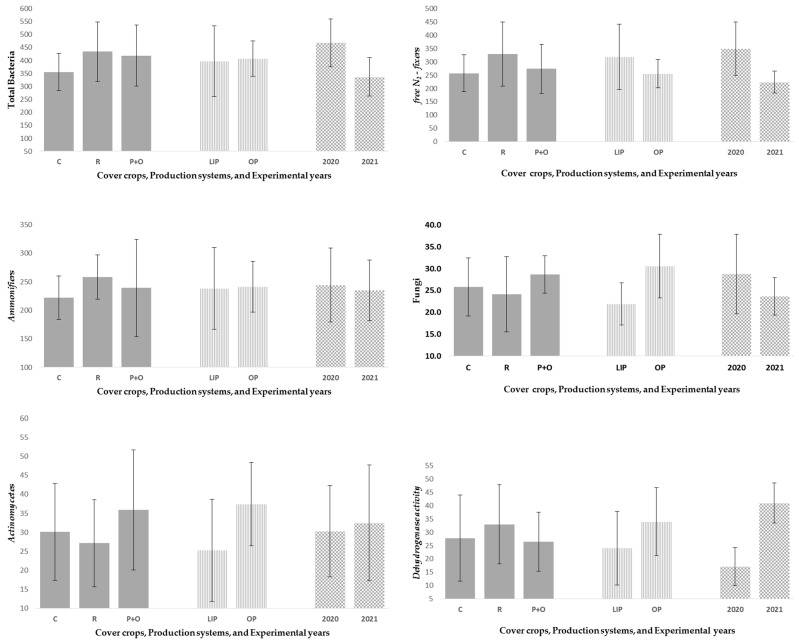
Average number of microorganisms (CFU g^−1^ soil (colony forming units)) in soybean rhizosphere during two-year trials (tb.—total bacteria × 10^7^; nfb.—free N_2_ fixers × 10^6^; amn.—ammonifiers × 10^6^; fng.—fungi × 10^4^; act.—actinomycetes × 10^4^) and dehydrogenase activity (μg TPF g^−1^ dry soil h^−1^) in soybean rhizosphere using different CCs and production systems. C—control; R—rye; P + O—peas and oats; LIP—low-input production; OP—organic production; 2020 and 2021—experimental years. Error bars show the standard deviation.

CCs also positively affect the soil microbial community structure and microbial properties and processes [27], which was confirmed in this study (Table 3). Our results indicate a significant increase in the abundance of ammonifiers and free N_2_-fixing bacteria in CC treatments in succession to soybean compared to the control, depending on the production system and the selected CC (Figure 6, Table 4).

**Figure 7:**
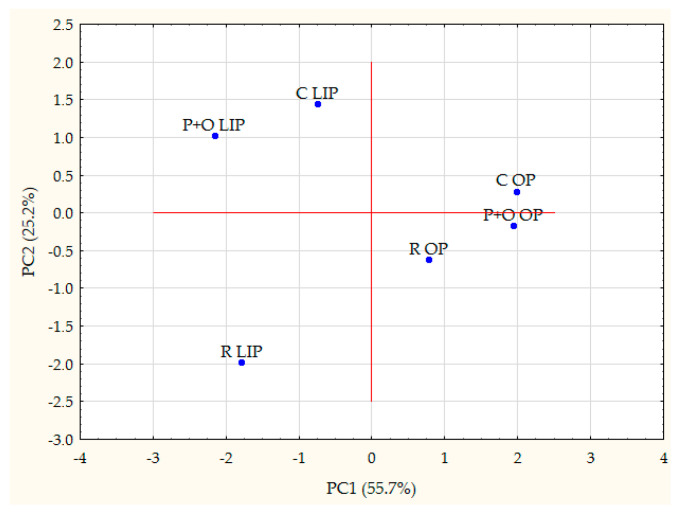
Principal component analysis (PCA) for the effects of different cover crops and production systems on the rhizosphere microorganisms in the first year (2020). C LIP—control in low-input production; C OP—control in organic production; R LIP—rye in low-input production; R OP—rye in organic production; P + O LIP—peas and oats in low-input production; P + O OP—peas and oats in organic production.

**Figure 8:**
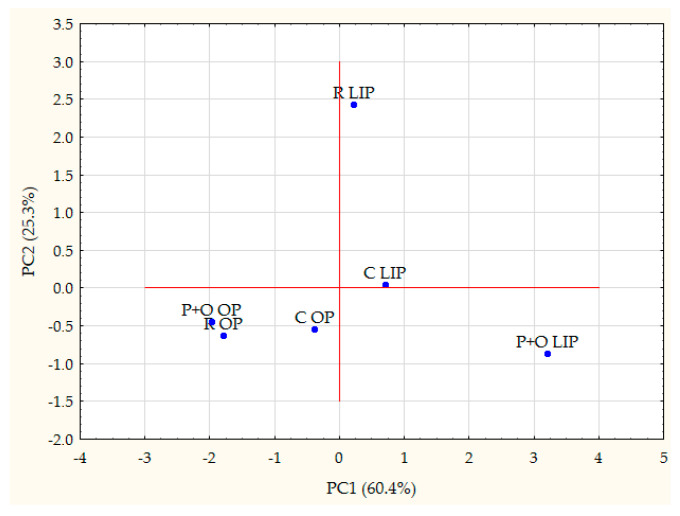
Principal component analysis (PCA) for the effects of different cover crops and production systems on the rhizosphere microorganisms in the second year (2021). C LIP—control in low-input production; C OP—control in organic production; R LIP—rye in low-input production; R OP—rye in organic production; P + O LIP—peas and oats in low-input production; P + O OP—peas and oats in organic production.

Figure 7 and Figure 8 present principal component analysis (PCA) of how various CCs and production systems affected the rhizosphere microorganisms. Strong differentiation among the different physiological groups of rhizosphere microorganisms across the CCs and production systems was observed. With 55.7% and 25.2% in Figure 7 and 60.4% and 25.3% in Figure 8, the two principal components (PC1 and PC2) account for a sizable amount of the variation.

## 3. Discussion

The major challenge in sustainable crop production is finding and testing new methods that have the potential to improve crop yield and nutritional quality [28]. The use of organic fertilizers and rotations that include CCs and legumes are some examples of these practices [29]. According to Ref. [30], a system of production that involves winter CCs without sacrificing a cash crop can be challenging to establish. Soybean in a system of winter CCs does not reduce yields and is a crucial management strategy to stop soil erosion, enable soil biota activity, and increase soil fertility over time [31]. This is in line with Ref. [32], who found that, when proper management practices were followed, CCs did not affect soybean growth and yield. In recent years, a decline in the frequency and intensity of low temperatures has been observed. In addition, the increased water consumption of CCs could be a disadvantage of their introduction. Consequently, it is essential to carefully manage CC termination times in order to reduce the competition for water resources with the main crop. The results of our experiment showed that soybean yield in succession to P + O as a winter CC was 3.0 t ha^−1^, whereas in succession to R as a CC, the yield was 2.7 t ha^−1^, and in the control, the soybean yield was 2.6 t ha^−1^. These results are lower than the results obtained by Ref. [33], where, over the years, the soybean yields varied from 3.0 to 4.5 t ha^−1^. Rainfall during the vegetation season has a major impact on soybean output, regardless of the presence of CCs. Our findings are in line with those of Ref. [34], who observed a similar trend that the yield from the treatment without CCs was lower compared to the treatment with CCs. LIP and OP rely more on the better management of on-farm resources, which leads to more sustainable practices because they depend less on input from outside of the farm [16]. According to Ref. [35], in organic production, soybean yields range from 673 to 3154 kg ha^−1^ and they are directly influenced by different management techniques. Also, proper selection of the varieties and hybrids of CCs, selected for the specific environmental conditions, methods, and goals of production, is one of the most important prerequisites for success in production [36]. Crop diversity is fundamental to enabling system sustainability [37].

**Figure 9:**
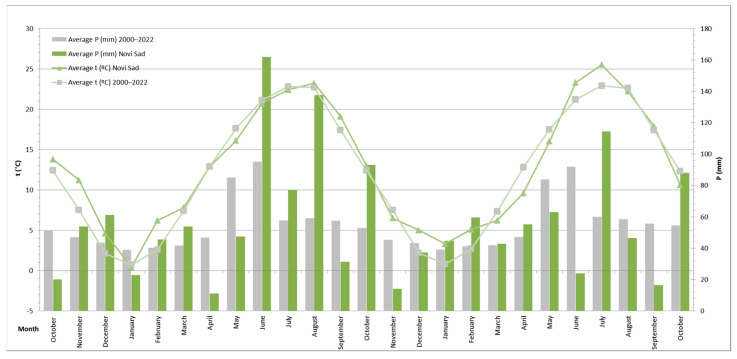
Average temperature (t °C) and precipitation (P mm) during the studied period (October 2019–October 2021) in comparison with the multi-year average (2000–2022).

Despite the multiple benefits of long-term CCs, in the short term, CCs could result in a shortage of available water for the cash crop [38]. In both examined years, higher precipitation than the multi-year average (Figure 9) during the soybean critical growth periods (beginning bloom (R1) to beginning pod (R3)) was noted. This is in line with the results of [39], who found that precipitation during the early growing season, which ran from April to July, was one of the main factors affecting the yields of soybeans. According to Ref. [40], planting rye as a CC has the advantage of preventing the leaching of leftover nitrogen, while legume species fix nitrogen to produce more nitrogen that will be utilized by the main crop. The C/N ratio of pea as a leguminous CC is lower than that of rye as a non-leguminous CC, and the mineralization of biomass is directly linked to the availability of nutrients to the main crop. Comparing both production systems, the soil parameter values for OP were much higher and the levels of phosphorus and potassium lower than in LIP. According to Ref. [41], the phosphorus and potassium built up during conventional management are exploited by organic mixed arable systems. The efficacy of CCs is dependent on the planting date, rate of N accumulation over the fallow season, N mineralization, and regional conditions [42].

As shown in Figure 1, the differences in soybean yield between the two CCs (P + O and R) were seen only in P + O. In addition, according to Ref. [43], selecting the right cover crop technique leads to the positive economic effects of CCs in organic soybean cultivation. Statistical differences between the two tested sustainable crop production systems revealed that OP had higher soybean yields in comparison with LIP. This can be explained by the availability and regular application of manure to the organic plots (not before the soybeans). The difference between the soybean yields in the different cultivation systems supports our hypothesis that winter CCs increased soybean yield compared to the control (C). The results are the opposite of those of Ref. [44], which revealed a non-significant effect on the yield, directly related to the selection of plant species as CCs. According to Ref. [45], a two-year period may be insufficient to obtain a direct effect of CCs; however, our results suggest positive effects of P + O as a CC on soybean yield.

For agriculture producers, data on soybean seed composition can be useful, especially from trials conducted in a wide range of target environments, including dry environments [46]. The obtained values of protein and oil contents are in accordance with the statements of [47], i.e., the results of the soybean protein and oil contents of organic and low-input macro trials in Serbia during 2021–2022. In comparing two sustainable production systems, it was observed that OP had higher oil content, and LIP had higher protein content (Figure 4 and Figure 5). Low input is an adequate production system if there are no opportunities for organic certification and for producers who are aware of the advantages of sustainable systems, and it can also represent a transitional path towards regenerative agriculture or organic production. In addition, in almost all aspects of the study, organic production was ahead of the low input; one of the reasons for this is the reflection of the soil parameters and differences in humus and nutrient content and microbial activity in both tested production systems.

It is crucial to determine the soil health indicators, especially the soil biogenicity, in which the potential of sustainable production systems is reflected [48]. The application of organic fertilizers or the sowing of CCs increases the availability of carbon to microorganisms [49]. A better understanding of how selected cropping systems influence soil microbial communities could be the main driver of further development of sustainable agricultural practices [17]. However, soil microbial communities, including their interactions, have received little attention in the testing of winter CCs [50]. The microbial activity in the soil is positively impacted by CCs; however, as microbial activity is correlated with abundance and diversity, this link needs to be interpreted carefully [51,52]. The prevalence of specific microbial groups directs the processes to the synthesis or decomposition of inorganic and organic matter that enters the soil. Plants influence the rhizosphere via root exudates, thereby contributing to the formation of host-specific microbial communities that are more abundant than in bulk soil [53]. Examined physiological groups of rhizosphere microorganisms in this study are important indicators of decomposition of organic matter, the cycling of nutrients, and many other activities essential to plant growth and development and ecosystem function [54]. Ammonifying bacteria degrade organic N compounds, whereas free nitrogen-fixing bacteria reduce atmospheric N_2_, converting it into forms available for plants. Fungi and actinomycetes are included in the cycles of main plant nutrients. They also produce a large number of enzymes necessary for the decomposition of complex organic compounds (cellulose, lignin, pectin, etc.) and participate in the synthesis of humus. Dehydrogenases are the constitutive enzymes of all microorganisms, and, based on their activity, the overall microbiological activities of the soil can be assessed.

The average number of rhizosphere microorganisms and dehydrogenase activity per different cover crops, production systems, and experimental years are listed in Figure 7. Cover crop composition is the main factor influencing cash crop root-associated microorganisms [55]. Moreover, CCs led to species- and even cultivar-specific bacterial and fungal shifts [56]. All three tested bacterial groups had the same pattern regarding the effect of CCs, with R, followed by P + O, being better than the control (Figure 5). Rye also had the best effect on dehydrogenase activity in rhizosphere soil (Figure 5). Similarly, different cover crops, particularly cereal rye and sorghum, selectively enrich specific microbial communities and their functions due to heterogeneity of root exudation [55]. The significant impact of CC factor on fungi and actinobacteria (Table 3), and the precedence of P + O treatment compared to others (Figure 6), implies that increased plant diversity led to a greater abundance of active decomposers and improved efficiency of C cycling. A higher rhizosphere carbon input stimulates microbial growth and activity, which has a beneficial effect on the growth and yield of plants [57]. Furthermore, total bacteria and ammonifiers were more abundant in OP treatment, although the differences between production systems were negligible. By contrast, the abundance of free nitrogen-fixing bacteria was significantly higher in the LIP than in the OP treatment. A positive effect of LIP management on free-living nitrogen-fixing communities in this study could be explained by the increased availability of phosphorus (P) in this treatment, which is indicated by the analysis of soil chemical properties before trial set up. For instance, Reed et al. [58] observed that the addition of P significantly improves nitrogen fixation due to high energy requirements for reducing atmospheric dinitrogen. This microbial group was also significantly affected by the CC, as well as the interaction of CC × PS factors (Table 3 and Table 4), which indicates that increased organic C, added through CCs, allows more optimal conditions for free nitrogen-fixing bacteria. Additionally, OP treatment led to a significant increase in the number of fungi and actinomycetes, as well as activity of dehydrogenase in the rhizosphere of soybean. This observation is consistent with other studies which showed that an increase in organic matter in organic production positively influences microbial abundance and activity [59]. In this study, the dynamics of rhizosphere microbial communities were also dependent on experimental year (Table 3 and Table 4, Figure 6). The composition and activity of bacterial and fungal communities significantly varied among seasons, representing one of the most reliable indicators of environmental changes [60]. Environment-dominated microorganisms are defined by the biological, chemical, and physical properties of the rhizosphere [61].

Our PCA (PC1 and PC2) indicates strong differentiation between the different physiological groups of rhizosphere microorganisms across the CCs and production systems (LIP and OP). Rye as a CC in low-input (R LIP) production is clearly separate from the other treatments, especially in PC1, indicating that R fostered a different microbial community than the control (C LIP) and the peas and oats (P + O LIP). These results suggest that the introduction of rye might have an influence on the rhizosphere microorganisms. On the other hand, in organic production, peas and oats (P + O OP) clustered near the control (C OP), indicating more similar microbial communities in these treatments. Moreover, rye (R OP) was positioned more distantly from the P + O OP and C OP treatments, indicating diversity in the microbial compositions. The position of peas and oats close to the control in OP contrasts with their distinct behavior in LIP, which suggests that the production system itself plays a key role in shaping the responses of rhizosphere microorganisms to cover crop management. The results of the PCA analysis across two experimental years differ despite the similar weather (Figure 9). In addition to the production systems and the cover crops, the observed changes in the rhizosphere could be explained by the complexity and impact of soil–plant–microbe interactions. Generally, the results indicate a significant increase in the number of ammonifiers, and free N_2_-fixing bacteria, depending on the production system and the selected cover crop. This is consistent with the results of Ref. [50], who found that abundance and diversity of soil microorganisms are significantly affected by different cover crop treatments.

## 4. Materials and Methods

**Scheme 1:**
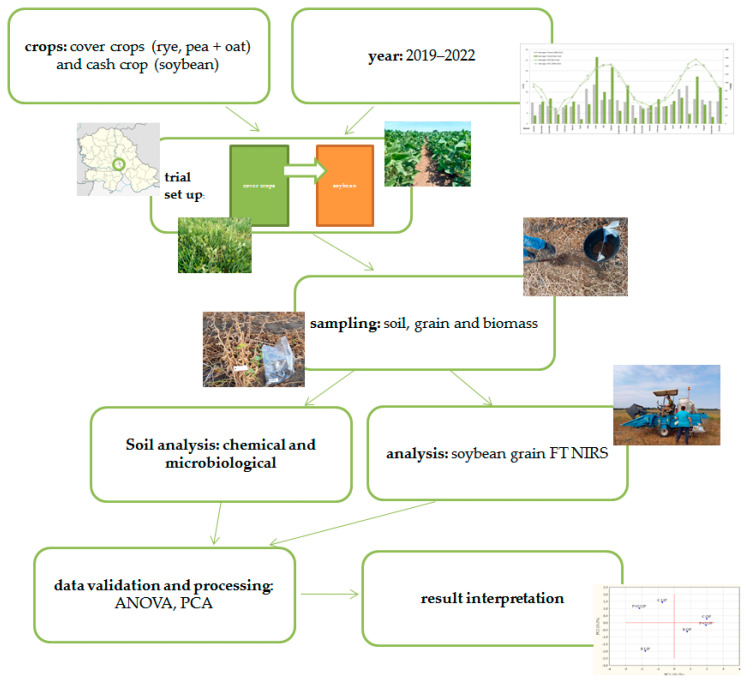
Dynamics of the experiment.

The trials were conducted under low-input production (LIP) and certified organic production (OP) during the period 2019–2021. The following scheme (Scheme 1, Supplementary Material, Table S1) presents the dynamics of the experimental set up, data collection, processing, and interpretation.

Weather conditions: The production year (2019/2020) was favorable for most of the field crops. During 2020 (April–October), the mean air temperature was 18.0 °C, and the amount of precipitation was 466.5 mm. Precipitation in June provided ideal conditions for a good yield and harvest of spring crops. The deeper soil layers were able to store moisture due to regular rainfall, and the plants used this valuable resource during the hot, dry summer months. In 2021 (April–October), the mean temperature and precipitation sum in the experimental fields were 17.9 °C and 407.1 mm, respectively. During 2021, the air temperature was below the multi-year average (2000–2022) in April and May, during sowing and plant emergence. In the subsequent months, the temperatures were above the long-term average, with higher average values in June and July. The precipitation was mostly lower during April and May 2021 than the multi-year average (2000–2022), especially in June and September, whereas July was the wettest month (Figure 9).

After the winter wheat harvest, in July, cool-season CCs were sown (LIP—27 October 2019 and 15 October 2020, OP—25 October 2019 and 2 October 2020), and soybean in 2020 and 2021 (Table 5). As a CC, pure rye crop (R) and a mixture of peas and oats (P + O) were sown, while the control treatment (C) was an area without CCs, where only soybeans were sown. The trials were set up according to a complete block design, with four replications.

The total area under trial was 30 × 90 m in both production systems (30 × 30 m per cover crop, 15 × 90 m per soybean variety). After mulching of the cover crop and disking, seedbed preparation was carried out, and, on the same day, soybeans were sown (LIP—24 April 2020 and 5 May 2021, OP—24 April 2020 and 3 May 2021). The soil type was a calcareous chernozem, CH-cc-ai.lo.ph (IUSS Working Group WRB, 2022). In the trial, two soybean varieties were tested: NS Mercury variety, 00 maturity group, and NS Altis variety, 0 maturity group, both developed at the Institute of Field and Vegetable Crops, Novi Sad, Serbia (Table 5). Cultivation practices were performed in accordance with the production system requirements: for LIP, combined use of organic and synthetic inputs and, for OP, use of organic inputs, certified production according to the EU organic production regulation. Weeds were controlled both mechanically and manually in both production systems.

Soil sampling and analysis: Prior to the experiment, composite soil samples were collected from the experimental fields for soil chemical analyses at a 0–30 cm soil depth. The collected soil samples were air-dried and milled to a particle size < 2 mm following the common soil chemical analyses method to determine soil chemical properties according to ISO 11464:2006 [62]. The soil chemical properties of the low-input and organic plots before the trial set up are available in Table 6.

Rhizosphere samples were taken from each treatment to determine the microbial abundance and activity. The soybean roots were carefully detached from the bulk soil, while the adhering soil was considered the rhizosphere. All rhizosphere samples were immediately placed in sterile polyethylene bags, transported to the laboratory, and stored in a refrigerator at 4 °C for subsequent microbial analyses [63]. Then, during both experimental years, the microbial abundance and dehydrogenase activity in the soybean rhizosphere were determined at full flowering (R2) and full maturity (R8), using the indirect dilution plate method [64] on appropriate nutrient media (Hi Media Laboratories Pvt. Limited, Mumbai, India). The microbial abundance included the total bacteria (soil agar), azotobacters and free N_2_ fixers (nitrogen-free medium), ammonifiers (nutrient agar), actinomycetes (synthetic agar), and fungi (Czapek-Dox agar). All microbiological analyses were performed in three replications, and the average number of microorganisms was calculated for 1.0 g of absolutely dry soil (CFU g^−1^ soil) [65]. In addition, the microbial activity was analyzed spectrophotometrically (Agilent Cary 60, Agilent Technologies, Santa Clara, CA, USA) by determining the dehydrogenase (DHA) activity. The activity of DHA (EC 1.1.1.) was determined by measuring the extinction of colored triphenylformazan (TPF) formed by reducing a colorless 2,3,5-triphenyltetrazolium chloride (TTC) [66].

Soybean grain and biomass sampling and analysis: The harvest was performed on 10 m^2^ of each treatment in four replications, the grain moisture was measured during the sample scaling, and the obtained values were transformed to tons per hectare (t ha^−1^) (based on a 14% moisture content). Soybean grain samples were taken to determine the protein and oil contents from each treatment in four replications. The total protein and oil contents of the soybean were determined using Fourier transform near-infrared spectroscopy on an Antaris II FT-NIR device, Thermo Scientific (Waltham, MA, USA). The FT-NIRS is a non-destructive fast technique capable of analyzing organic substances, which, in this particular case, was soybean grain. OMNIC^TM^ software, Version 9 was used for data processing and calibration. In addition, plant height (cm) and biomass (g) were measured in four plants per treatment in four replications, randomly selected from inner rows to avoid the edge effect.

Data processing: The collected data were analyzed in accordance with the experimental design. In the trial, four factors were defined (winter cover crop, production system, soybean variety, and experimental year). The data were statistically processed in R (4.3.2) and (StatSoft Inc., Tulsa, OK, USA) using the analysis of variance (ANOVA) statistical method, followed by mean separation according to Tukey’s HSD test (*p* < 0.05) and principal component analysis (PCA).

## 5. Conclusions

One of the main challenges for agricultural producers is the question of how to introduce cover crops to existing crop rotation schemes of the most important field crops and how much this system of production will affect the ultimate success of production. Many studies have observed how the introduction of legumes, e.g., soybeans, influences changes at the crop rotation level. In this study, the perspective was different: how can winter CCs influence soybean production as a cash crop under sustainable production schemes? The hypothesis that winter CCs would improve soybean yield was supported by a significant increase in the soybean yields between the tested P + O as a CC and the control, and the types of production system (LIP or OP) were observed. In our study, there were no barriers to legumes (peas) being grown as a CC for soybeans. The results showed increased abundance and activity of microorganisms in the soybean rhizosphere, which primarily depended on production system and selected CCs. The findings of this study can be a keystone for production improvement in the sustainability dimension in regions throughout Southeast Europe, where a decline in crop rotational diversity has been seen, especially in soybean production. Low-input farming can be considered a transition pathway toward organic farming or regenerative agriculture. Long term, CCs can be the answer to concrete actions toward agro-biodiversity, along with other benefits that CCs can provide at the level of crop rotation.

## Figures and Tables

**Table 1:** Analysis of variance for soil yield, height, and plant biomass under different cover crops and production systems in two-year trials.

Factors	Variables		
	Yield	Height	Biomass
Cover crops (CC)	0.000000 ***	0.933153	0.000371 ***
Production system (PS)	0.000001 ***	0.000000 ***	0.889682
Year (Y)	0.002694 **	0.000000 ***	0.000009 ***
CC × PS	0.861339	0.458956	0.007894 ***
CC × Y	0.318464	0.801140	0.169923
PS × Y	0.621353	0.000001 ***	0.000432 ***
CC × PS × Y	0.260262	0.000294	0.137627

** *p* ≤ 0.01; *** *p* ≤ 0.001; without *—not significant.

**Table 2:** Analysis of variance for protein and oil contents under different cover crops and production systems in two-year trials.

Factors	Variables	
	Protein	Oil
Cover crops (CC)	0.265856	0.197641
Production system (PS)	0.013611	0.000008 **
Year (Y)	0.597864	0.050979
CC × PS	0.872744	0.731202
CC × Y	0.984607	0.992290
PS × Y	0.021296	0.000062 **
CC × PS × Y	0.757017	0.736991

** *p* ≤ 0.01; without *—not significant.

**Table 3:** Analysis of variance for rhizosphere microorganisms under different cover crops and production systems in two-year trials.

Factors	Variables					
	Total Bacteria	Ammonifying Bacteria	Nitrogen-Fixing Bacteria	Fungi	Actinobacteria	Dehydrogenase
Cover crops (CC)	0.0018 **	0.1106	0.0003 ***	0.0277 *	0.0346 *	0.0000 ***
Production system (PS)	0.5463	0.8451	0.0000 ***	0.0000 ***	0.0001 ***	0.0000 ***
Year (Y)	0.0000 ***	0.5178	0.0000 ***	0.0005 ***	0.4135	0.0000 ***
CC × PS	0.4802	0.6596	0.0304 **	0.1132	0.0968	0.0589
CC × Y	0.0827	0.0146 *	0.0051 **	0.1052	0.2627	0.0000 ***
PS × Y	0.0000 ***	0.0002 ***	0.0000 ***	0.0000 ***	0.0000 ***	0.6969
CC × PS × Y	0.0776	0.0066 ***	0.0550	0.2371	0.0299 **	0.0061 *

* *p* ≤ 0.05; ** *p* ≤ 0.01; *** *p* ≤ 0.001; without *—not significant.

**Table 4:** Tukey’s HSD test for observed microorganisms in soybean rhizosphere.

Cover Crops	Production System	Year	TB	AMN	NFB	FNG	ACT	DHA
Control	Low input	2020	430 a–e	223 bc	352 bc	19.9 d	36.9 a–e	6.9 f
		2021	296 d–f	204 bc	240 c–e	25.6 b–d	20.7 c–e	41.7 b
	Organic	2020	359 c–f	194 bc	235 de	32.9 a–c	26.2 b–e	18.7 de
		2021	334 c–f	265 a–c	199 e	24.9 b–d	39.9 a–d	43.3 b
Rye	Low input	2020	569 a	252 a–c	506 a	19.9 d	17.2 de	16.4 de
		2021	283 ef	258 a–c	251 c–e	15.7 d	18.7 de	39 b
	Organic	2020	472 a–c	247 a–c	340 b–d	36 ab	28.8 b–e	23.6 cd
		2021	410 b–e	275 ab	221 e	24.7 b–d	43.9 ab	52.3 a
Peas + Oats	Low input	2020	549 ab	344 a	398 ab	23.9 cd	41.8 abc	12.5 ef
		2021	254 f	149 c	161 e	26.4 b–d	16.2 e	27.7 c
	Organic	2020	430 a–e	205 bc	267 c–e	39.9 a	30.8 b–e	24.2 cd
		2021	439 a–d	260 a–c	266 c–e	24.4 cd	55 a	41.3 b

Data are presented as the means (*n* = 3). Different letters in the same column denote statistically significant differences (*p* ≤ 0.05, of Tukey’s HSD test). Note: TB—total number of bacteria × 10^7^; AMN—number of ammonifying bacteria × 10^6^; NFB—number of free nitrogen-fixing bacteria × 10^6^; FNG—fungi × 10^4^; act.—ACT × 10^4^, (CFU g^−1^ soil); DHA—dehydrogenase activity (μg TPF g^−1^ dry soil h^−1^) in soybean rhizosphere.

**Table 5:** General information about trial sowing at both locations.

Period	Crop (Variety)	Seed Amount
October 2019/2020	Rye (NS Savo)	210 kg ha^−1^
October 2019/2020	Mixture (85:15):	
	Pea (Kosmaj)	140 kg ha^−1^
	Oat (NS Jadar)	30 kg ha^−1^
April 2020/May 2021	Soybean (NS Mercury)	100 kg ha^−1^
	Soybean (NS Altis)	95 kg ha^−1^

**Table 6:** Soil chemical properties of low-input and organic plots (0–30 cm) before trial set up.

Production System	pH		CaCO_3_ %	Humus %	Total N %	AL-P_2_O_5_ mg/100 g	AL-K_2_O mg/100 g
	KCl	H_2_O					
LIP *	7.4	8.2	5.9	2.3	0.2	17.9	20.9
OP **	7.5	8.4	13.7	3.7	0.3	7.2	15.0

* LIP—low-input production, ** OP—organic production.

## Data Availability

Data supporting reported results can be found at https://doi.org/10.5281/zenodo.13772851.

## Supplementary Materials

The following supporting information can be downloaded at: https://www.mdpi.com/article/10.3390/plants13213091/s1. Table S1: Trial Layout.
