# Supplementary material for: Winter Cover Cropping in Sustainable Production Systems: Effects on Soybean and Synergistic Implications for Rhizosphere Microorganisms
Source: Plants (Basel). 2024 Nov 2;13(21):3091. doi: 10.3390/plants13213091 (PMC11548640; doi:10.3390/plants13213091)
Supplement: Supplementary file 1 [file plants-13-03091-s001.zip › plants-3261730-supplementary.pdf]

Table S1: Trial layout

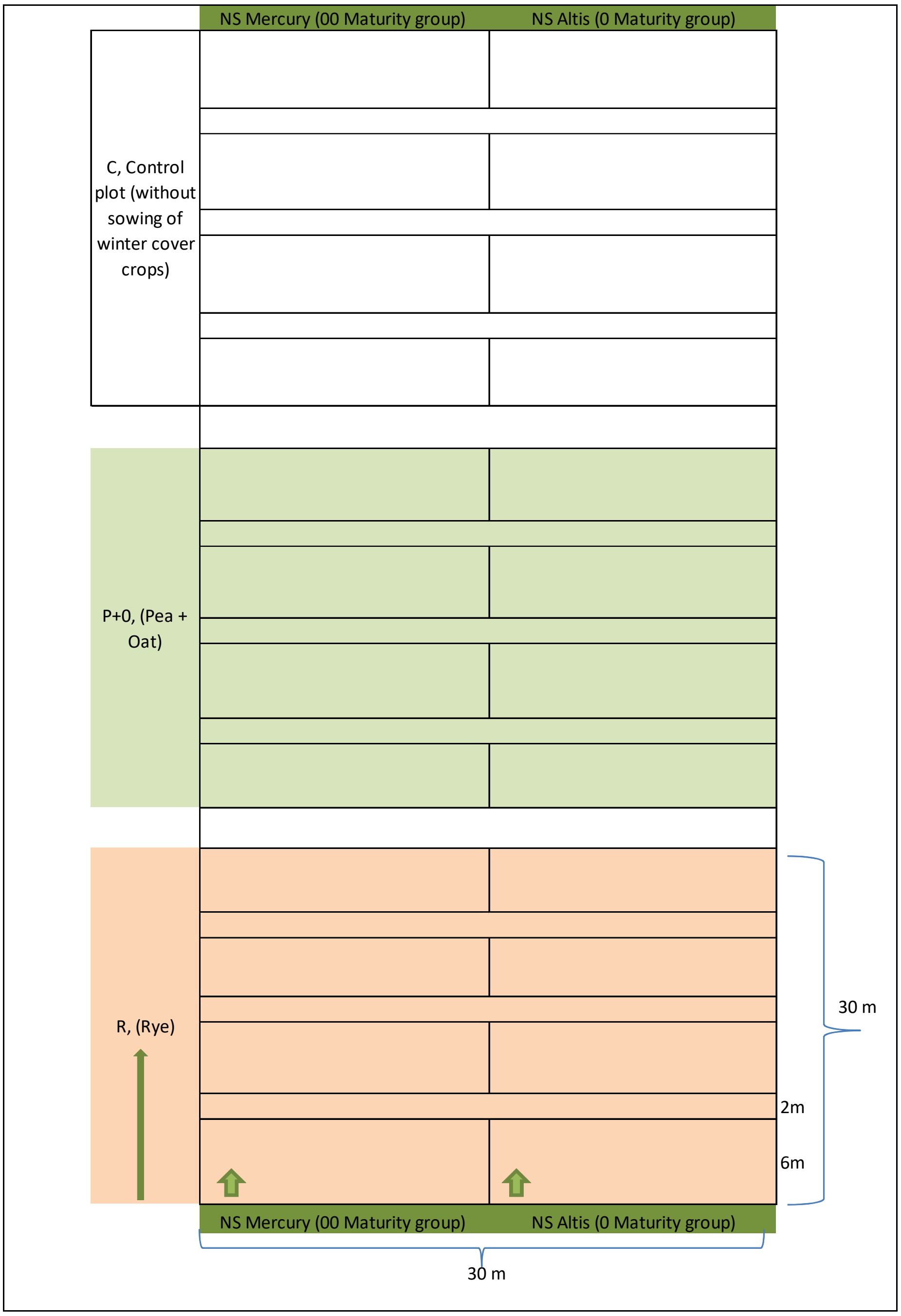

**Factors:**	Winter Cover Crops	Rye (R), Pea + Oat (P+O), C (control without sowing of winter cover crops)
	Production Systems	Low input and organic production
	Soybean Variety	NS Mercury and NS Altis
	Experimental Year	2019. sowing of winter cover crops
		2020. first season of soybean sowing
		2020. sowing of winter cover crops
		2021. second season of soybean sowing
**Note:**	The total area under trial was 30 × 90 m in both production systems (30 × 30 m per cover crop, 15 × 90 m per soybean variety).	
